# *ApoE*/*NOS3* Knockout Mice as a Novel Cardiovascular Disease Model of Hypertension and Atherosclerosis

**DOI:** 10.3390/genes13111998

**Published:** 2022-11-01

**Authors:** Ke Liu, Bangzhu Chen, Fanwen Zeng, Gang Wang, Xin Wu, Yueshu Liu, Guiling Li, Jiarong Yan, Shouquan Zhang

**Affiliations:** 1 National Engineering Research Center for Breeding Swine Industry, Guangdong Provincial Key Laboratory of Agro-Animal Genomics and Molecular Breeding, College of Animal Science, South China Agricultural University, Guangzhou 510642, China; 2 Guangdong Medical Laboratory Animal Center, Foshan 528248, China

**Keywords:** double gene knockout, *ApoE*, *NOS3*, atherosclerosis, hypertension, effect assessment

## Abstract

Hypertension is an independent risk factor for atherosclerosis. However, few models of hypertensive atherosclerosis have been established in medical research. In this study, we crossed the *ApoE* knockout (*ApoE*-KO; *ApoE^−/−^*) atherosclerotic mouse model with the *NOS3* knockout (*NOS3*-KO; *NOS3^−/−^*) hypertensive mouse model to establish an *ApoE/NOS3* double knockout (*ApoE/NOS3*-KO; *ApoE/NOS3^−/−^*) hypertensive atherosclerosis mouse model. We found that *ApoE/NOS3^−/−^* mice reproduced normally, had a blood pressure of 133.00 ± 3.85 mmHg, and developed hypertensive fundus retinopathy and hypertensive nephropathy. In addition, serum total cholesterol (TC) and low-density lipoprotein (LDL) levels in the blood were abnormally elevated, steatosis was observed in the liver cells, and atherosclerotic lesions were observed in the aortic vessels in *ApoE/NOS3^−/−^* adult mice. In conclusion, *ApoE/NOS3^−/−^* adult mice are a satisfactory model of hypertension and atherosclerosis and can be utilized for studies on cardiovascular diseases.

## 1. Introduction

Atherosclerosis is the leading cause of death due to cardiovascular diseases worldwide [1]. This pathological disease is characterized by vascular wall fiber proliferation, chronic inflammation, lipid accumulation, and immune disorders [2,3,4]. With the development of atherosclerotic plaques in the late stage, vulnerable plaques are prone to rupture, leading to acute cardiovascular events, including ischemic stroke and myocardial infarction [5,6]. Atherosclerosis is related to multiple factors, including a family history of cardiovascular and cerebrovascular diseases, age, sex, hypercholesterolemia, diabetes, hypertension, smoking, and obesity [7]. Although hypertension is not the most common risk factor for atherosclerosis, it is the major independent risk factor associated with atherosclerotic cardiovascular disease because of its high prevalence [8,9]. Studies have shown that long-term hypertension commonly causes atherosclerotic lesions [8,10].

Apolipoprotein E (ApoE) is component of chylomicrons, very-low-density lipoproteins, and intermediate-density lipoproteins. It plays an important role as a ligand in receptor-mediated lipoprotein uptake. ApoE acts as a ligand for low-density lipoprotein (LDL) receptors and LDL receptor-related proteins [11]. Piedrahita et al. first obtained the mouse model of atherosclerosis in 1992 by inactivating the *ApoE* gene and feeding mice a high-fat diet [12]. Subsequently, Ishibashi et al. developed a double knock-out model lacking both the *ApoE* gene and the LDL receptor, which is most frequently used to analyze complex atherosclerotic lesions [13,14]. Currently, *ApoE* knockout (*ApoE*-KO; *ApoE^−/−^*) mice are commonly used to study human atherosclerosis; however, they lack the typical symptoms of hypertension and poorly mimic human hypertension-induced atherosclerosis [15]. Therefore, establishing an ideal model for research on hypertensive atherosclerosis is necessary.

Hypertension is a major risk factor for cardiovascular diseases, including myocardial infarction and stroke [16]. Hypertension can cause local endothelial dysfunction that promotes the entry of lipids into the vessel wall and the subsequent formation of lipid-rich fatty streaks, which are the main symptoms of “early” atherosclerotic lesions [17]. The main determinants of blood pressure are vasodilator factors, such as nitric oxide (NO) released from the endothelium under the influence of fluid shear stress imposed by blood flow through the vessels [18]. Particularly in the presence of hypertension, NO inhibits the hypertrophy and proliferation of mesangial cells and the synthesis of the extracellular matrix as well as regulates the hypertrophy and proliferation of smooth muscle cells [19]. Endothelial nitric oxide synthase 3 (NOS3), a member of the nitric oxide synthase (NOS) family, promotes vasodilation and participates in the regulation of angiogenesis. The restriction or promotion of *NOS3* expression leads to unstable NO production and the damage and dysfunction of the vascular endothelium, leading to diseases, such as atherosclerosis and hypertension. The dysfunction of the endothelial NOS gene can cause hypertension and impair salt excretion [20,21]. 

To obtain a better model of the relationship between hypertension and atherosclerosis, we used the *ApoE^−/−^* atherosclerotic mouse model and the *NOS3* knockout (*NOS3*-KO; *NOS3^−/−^*) hypertensive mouse model to cross-backcross and establish an *ApoE/NOS3* double knockout (*ApoE/NOS3*-KO; *ApoE/NOS3^−/−^*) mouse model of hypertensive atherosclerosis. We found that *ApoE/NOS3^−/−^* mice had a typical hypertensive phenotype and a more severe atherosclerotic phenotype than *ApoE^−/−^* mice. In addition, *ApoE/NOS3^−/−^* mice demonstrated normal fertility and could normally produce offspring, which is extremely convenient for researchers. This study provides a new model for further research on cardiovascular disease.

## 2. Materials and Methods

### 2.1. Generation of ApoE and NOS3 Double Gene Knockout Mice

*ApoE^−/−^* mice (murine strain: B6.129P2-Apoetm1Unc/J, JAX:002052) and *NOS3^−/−^* mice (murine strain: B6.129P2-Nos3tm1Unc/J, JAX:002684) were retrieved from the Jackson Laboratory in the United States. *ApoE^−/−^* mice were crossed and backcrossed with *NOS3^−/−^* mice, and *ApoE/NOS3^−/−^* mice were obtained through genotype identification and screening. The corresponding genotype identification sites and methods were obtained from the Jackson Laboratory website (https://www.jax.org/cn/, accessed on 1 January 2013). The diets of the experimental animals were as follows: (1) chow diet group, mice were fed a basal diet containing 4.5% fat; (2) obesogenic diet group, mice were fed a 10% high-fat diet before 8 weeks of age and a 21% high-fat diet after 8 weeks of age. Animal diets were provided by the Guangdong Provincial Medical Laboratory Animal Center. All experimental protocols were approved by the Ethics Committee for Animal Experiments of the Guangdong Medical Laboratory Animal Center, China (ethical review nos. B201607-4, B201609-12).

### 2.2. Measurements of Systolic Blood Pressure 

Systolic blood pressure was measured using a computerized tail-cuff system (BP2010A; Softron, Beijing, China). The readings were taken in separate rooms at the same time of day at a room temperature of 20–24 °C. Mice were placed in restrainers, with tails placed through a cuff, and onto a heart rate monitor. The entire procedure was performed gently to avoid stressing the mice. After the mice were quiet in the restrainers, a preliminary consecutive stable blood pressure measurement was performed to ensure that the blood pressure was detectable. At least three measurement cycles were performed to measure the correct systolic blood pressure and calculate the mean value.

### 2.3. Ophthalmoscope

The tropicamide mydriatic agent was instilled in both eyes of the mice. After 2 min, the eyes were topically anesthetized through the instillation of proparacaine hydrochloride. After 5–10 min, the mice were anesthetized with 1% pentobarbital (50 mg/kg). A retinal imaging microscope (Micron IV, Phoenix Research Labs, Phoenix, AZ, USA) was used to observe the retinal fundus. Drops were then added to keep the eyes moist.

### 2.4. Hematoxylin-Eosin (H&E) Staining

The mice were euthanized after anesthesia according to laboratory animal practice standards. The mice were then dissected to extract the tissue, which was fixed in a 4% paraformaldehyde solution and washed in phosphate-buffered saline for 4 min. Subsequently, the tissues were dehydrated using a graded ethanol series (75% ethanol for 4 h, 85% ethanol for 2 h, 90% ethanol for 1 h, 95% ethanol for 1 h, and 100% ethanol for 0.5 h) and embedded in paraffin for sectioning. Then, 5 μm tissue sections were mounted on siliconized slides. Sections were first dewaxed with xylene (10 min); successively dewaxed in 100% ethanol, 95% ethanol, 85% ethanol, and 70% ethanol for 5 min for each; and transferred to distilled water. Subsequently, the sections were stained in Mayer’s hematoxylin for 15 min and rinsed with distilled water before differentiation in 0.5–1% hydrochloric acid ethanol for 20 s. These sections were then stained with eosin dye for 3 min, and rinsed with distilled water. The slides were dehydrated in 85%, 95%, and 100% ethanol for 2 min each, washed twice with xylene for 5 min each time, and finally, sealed with neutral resin. Pathological sections were observed under a microscope, and images were obtained.

### 2.5. Serum Lipid Profiling

After a 12 h fast, mice were anesthetized with pentobarbital, enucleated to collect whole blood, and euthanized. Whole blood was allowed to stand at room temperature for 15 min and then centrifuged at 3000× *g* rpm and 4 °C for 5 min to separate the serum. Total cholesterol (TC), triglyceride (TG), LDL, and high-density protein (HDL) levels were measured using a Hitachi7600 Automatic Biochemistry Analyzer.

### 2.6. Quantification of Atherosclerosis

After the mice were euthanized via overdosing with anesthesia, the thoracic aortic root connected to the heart was harvested. The aortas were fixed in 4% paraformaldehyde for 24 h and dehydrated with 30% sucrose for more than 48 h. Finally, the aortas were embedded with OCT at −25 °C and operated according to the frozen pathological section procedure. Atherosclerotic lesions were quantified using a cross-section of the aortic root. The thickness of each frozen section was 8 μm. Six sections were collected for each sample. Images were obtained using a microscope, and the atherosclerotic plaque area was analyzed and calculated using Image-Pro Plus 6.0.

### 2.7. Statistical Analysis

SPSS 22.0 software was used for the statistical analysis of the data. The means and standard deviations of the measured data were analyzed. All data are presented as means ± standard deviations (SDs). Comparisons between two groups were performed using independent *t*-test statistical analyses, and comparisons among multiple groups were performed using one-way statistical analyses of variance. A *p*-value > 0.05 was considered to have no statistical difference; a *p*-value < 0.05 was considered to have a statistical difference; and a *p*-value < 0.01 was considered statistically significant.

## 3. Results

### 3.1. ApoE/NOS3^−/−^ Mice with a Hypertensive Phenotype

**Figure 1:**
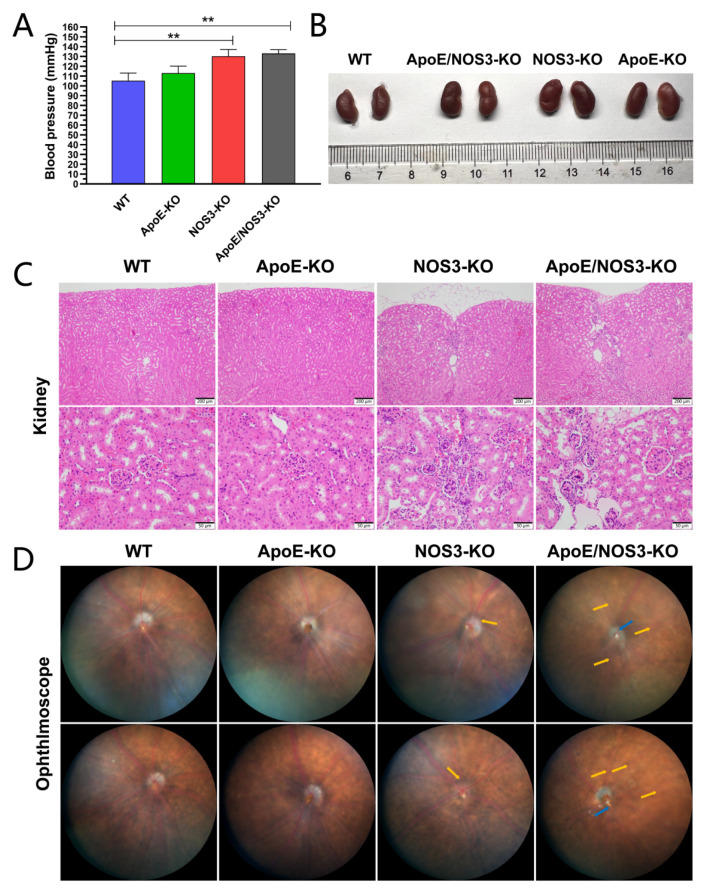
The hypertension phenotype of *ApoE/NOS3^−/−^* mice. (**A**) The blood pressure of WT, *ApoE^−/−^*, *NOS3^−/−^*, and *ApoE/NOS3^−/−^* mice. Data are presented as the means ± SEM. ** *p*-value < 0.01. (**B**) The kidneys of WT, *ApoE^−/−^*, *NOS3^−/−^*, and *ApoE/NOS3^−/−^* mice. Both *ApoE/NOS3^−/−^* and *NOS3^−/−^* mice developed different degrees of renal atrophy. (**C**) H&E staining of kidney tissue. The *ApoE/NOS3^−/−^* group and *NOS3^−/−^* group showed renal cortical atrophy, glomerular vacuolar lesions, glomerular interstitial fibrosis, and increased inflammatory cells. The scale bars for high and low magnification images are 200 μm and 50 μm, respectively. (**D**) Ophthalmoscope images. Compared with the WT, the fundus vessels of *NOS3^−/−^* and *ApoE/NOS3^−/−^* mice were narrowed (yellow arrows). Among them, *ApoE/NOS3^−/−^* mice had the most severe stenosis of fundus blood vessels, showing the cross-compression of blood vessels (blue arrows).

Blood pressure values in *ApoE/NOS3^−/−^* mice at 16 weeks of age were compared with those in age-matched C57BL/6 wild-type (WT) mice and *ApoE^−/−^* mice. The results showed that *ApoE/NOS3^−/−^* mice had a typical hypertensive phenotype, but *ApoE^−/−^* mice did not, and the blood pressure of *ApoE/NOS3^−/−^* mice reached a hypertensive level of 133 ± 3.85 mmHg (Table 1; Figure 1A). The blood pressures of WT and *ApoE^−/−^* mice of the same age were within the normal range. As shown in Figure 1B–D, *ApoE/NOS3^−/−^* mice had features of hypertensive nephropathy and hypertensive retinopathy. Overall, *ApoE/NOS3^−/−^* mice had more severe hypertensive complications than *NOS3^−/−^* mice.

### 3.2. ApoE/NOS3^−/−^ Mice Have a Dyslipidemia Phenotype

**Figure 2:**
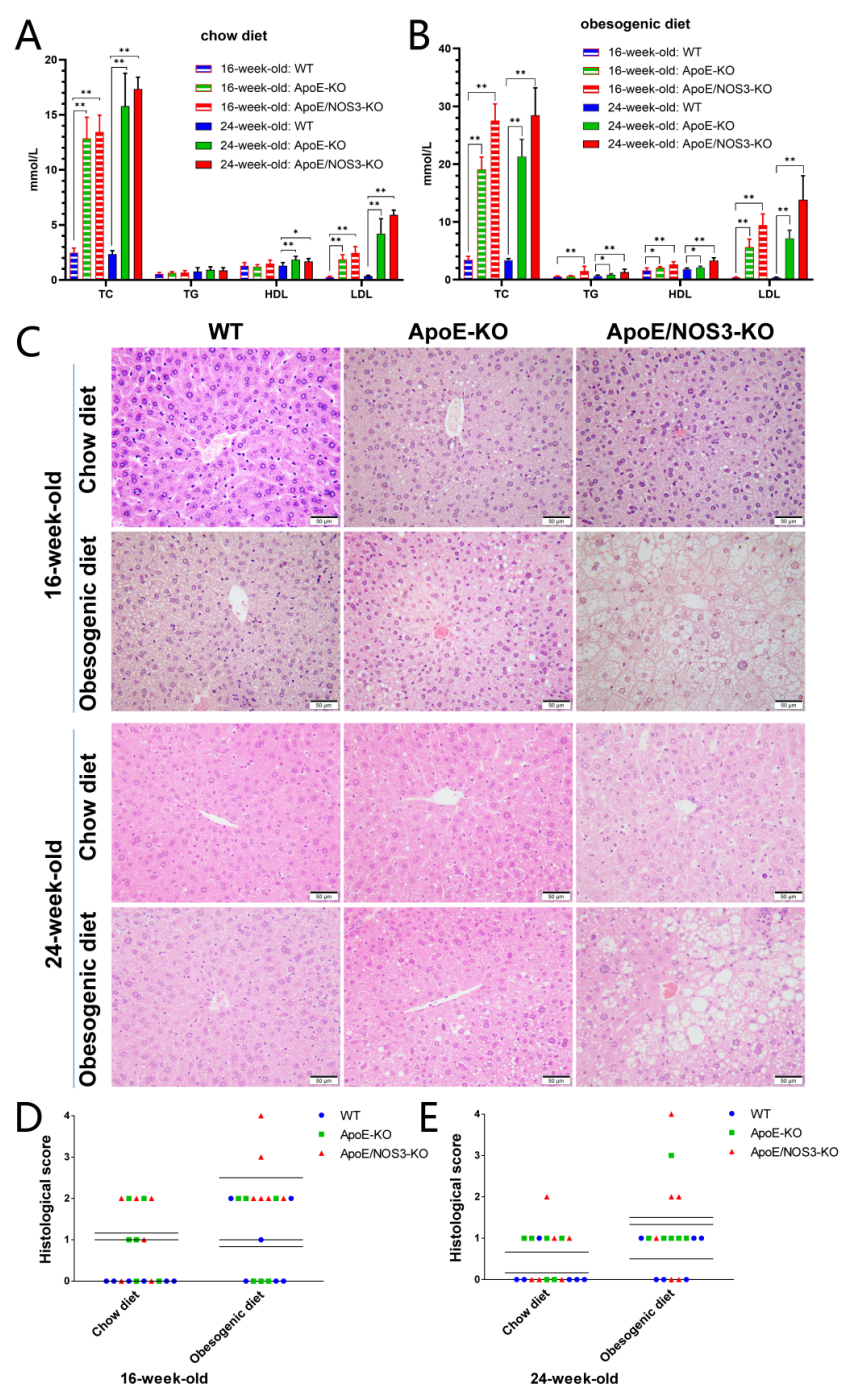
The lipidemia phenotype of *ApoE/NOS3^−/−^* mice. (**A**) The blood lipid levels of WT, *ApoE^−/−^*, and *ApoE/NOS3^−/−^* mice with a chow diet. TC, total cholesterol; TG, triglyceride; HDL, high-density protein; LDL, low-density lipoprotein. (**B**) The blood lipid levels of WT, *ApoE^−/−^*, and *ApoE/NOS3^−/−^* mice with an obesogenic diet. (**C**) H&E staining of WT, *ApoE^−/−^*, and *ApoE/NOS3^−/−^* mice. (**D**,**E**) Histological analysis of hepatocellular lesions. Scoring results: No abnormality was scored at 0 points, very mild at 1 point, mild at 2 points, moderate at 3 points, and severe at 4 points. Characteristics of the liver tissue lesions of WT, *ApoE^−/−^*, and *ApoE/NOS3^−/−^* mice fed with a chow diet or an obesogenic diet. *ApoE/NOS3^−/−^* mice had obvious hepatic steatosis. The scale bar is 50 μm. Data are presented as the means ± SEM. * *p*-value < 0.05; ** *p*-value < 0.01.

Blood lipids were measured and analyzed in WT, *ApoE^−/−^*, and *ApoE/NOS3^−/−^* mice fed a chow diet or an obesogenic diet (Table 2 and Table 3). The results showed that the TC and LDL levels of ApoE^−/−^ mice and ApoE/NOS3^−/−^ mice at 16 and 24 weeks of age in the normal and high-fat diet groups were significantly higher than those of WT mice in the same period (Figure 2A,B). *ApoE^−/−^* and *ApoE/NOS3^−/−^* mice have a hyperlipidemic phenotype, and *ApoE/NOS3^−/−^* mice have higher levels of TC and LDL in their blood than *ApoE^−/−^* mice. The pathological results of the H&E staining of liver tissue sections showed that *ApoE/NOS3^−/−^* mice had more severe liver lesions than WT and *ApoE^−/−^* mice of the same age (Figure 2C–E). Under special high-fat diet conditions, *ApoE/NOS3^−/−^* mice developed severe hepatocyte steatosis and hepatocyte enlargement.

### 3.3. ApoE/NOS3^−/−^ Mice with a Typical Atherosclerotic Phenotype

**Figure 3:**
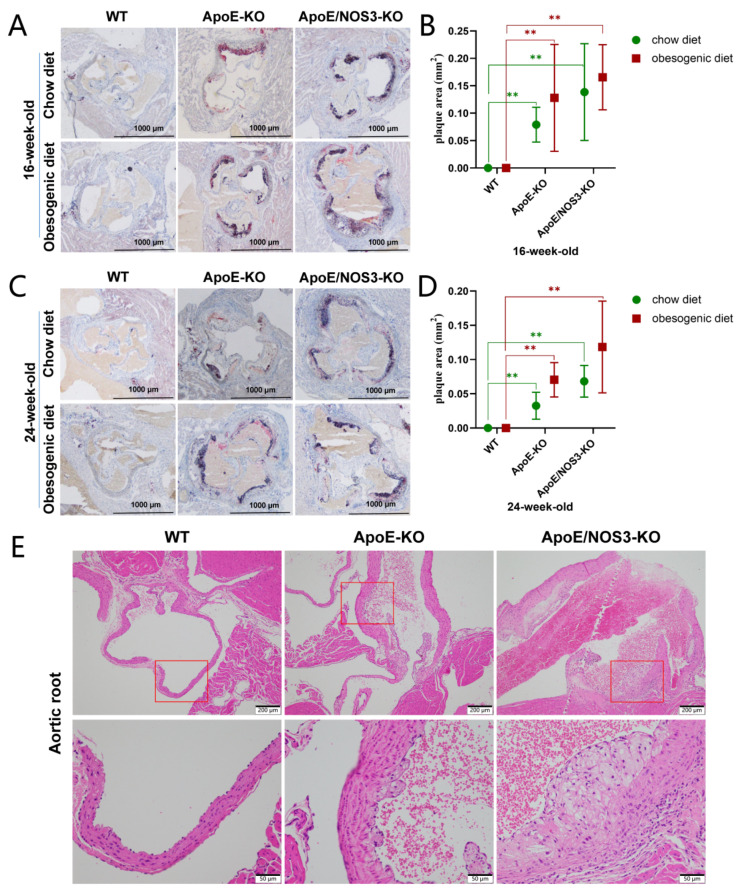
The typical atherosclerotic phenotype of *ApoE/NOS3^−/−^* mice. (**A**–**D**) Oil red O staining in the aortic root of WT, *ApoE^−/−^*, and *ApoE/NOS3^−/−^* mice. The scale bar is 1000 μm. Graphic (**B**) is the statistics of (**A**) results (*n* = 6), and graphic (**D**) is the statistics of (**C**) results (*n* = 6). *ApoE^−/−^* mice and *ApoE/NOS3^−/−^* mice have atherosclerotic plaque lesions, and *ApoE/NOS3^−/−^* mice have more severe lesions. Data are presented as the means ± SEM, ** *p*-value < 0.01. (**E**) H&E staining of the aortic root of WT, *ApoE^−/−^*, and *ApoE/NOS3^−/−^* mice. The red box is the field of view corresponding to the high-magnification image. The scale bars for high- and low-magnification images are 200 μm and 50 μm, respectively. High-magnification images show atherosclerotic lesions. *ApoE^−/−^* mice and *ApoE/NOS3^−/−^* mice have atherosclerotic plaque lesions, and *ApoE/NOS3^−/−^* mice have more severe lesions.

*ApoE^−/−^* and *ApoE/NOS3^−/−^* mice developed severe atherosclerotic plaques for the normal diet and the high-fat diet (Figure 3A–D). However, WT mice of the same age and diet did not develop atherosclerotic plaques. H&E staining results showed that *ApoE^−/−^* and *ApoE/NOS3^−/−^* mice had atherosclerotic plaque lesions, and in *ApoE/NOS3^−/−^* mice, these lesions had become severe (Figure 3E).

### 3.4. ApoE/NOS3^−/−^ Mice Can Reproduce Offspring from Homozygous Interbreeding

The *ApoE/NOS3^−/−^* mice that mated under normal dietary conditions produced more offspring that were homozygous for double gene knockout. The litter size and weaning number of the *ApoE/NOS3^−/−^* mice were not significantly different from those of *ApoE^−/−^* and *NOS3^−/−^* mice (Table 4). Notably, *ApoE/NOS3^−/−^* mice fed a high-fat diet had a higher rate of conception failure and a lower litter size than those fed a normal diet.

## 4. Discussion

Hypertension and atherosclerosis are among the most common causes of death worldwide. Therefore, experimental animal models of hypertension and atherosclerosis have become valuable tools for studying the etiology of the disease and the mechanisms of treatment and action [22,23,24]. The establishment of a stable mouse model of hypertensive atherosclerosis is important for studying cardiovascular diseases. ApoE has been identified as a major risk factor for cardiovascular disease and is correlated with an increased risk of atherosclerosis [25,26,27]. The most common phenotypes of *ApoE*-KO mice are hyperlipidemia and the spontaneous development of atherosclerosis [28]. The possible roles of oxidative stress and nitric oxide (NO) in the pathogenesis of multiple sclerosis have been established [29]. Recently, the literature has shown that oxidative stress-induced abnormalities in vascular NO production, transport, and metabolism result in endothelial dysfunction, contributing to various cardiovascular diseases, such as hypertension, atherosclerosis, and angiogenesis-related disorders [30,31,32]. *NOS3* knockout mice have also been associated with vascular function [33,34]. Therefore, in this study, we established an *ApoE* and *NOS3* double knockout mouse model with more similar clinical phenotypes in hypertensive atherosclerotic diseases.

In this study, we established an *ApoE/NOS3^−/−^* mouse model of hypertensive atherosclerosis. *ApoE/NOS3^−/−^* mice can reach a blood pressure of 133.00 ± 3.85 mmHg in adulthood and suffer from hypertension. *ApoE/NOS3^−/−^* mice develop hypertensive lesions in the kidneys and retinas. *ApoE^−/−^* mice had normal blood pressure levels, and *ApoE/NOS3^−/−^* mice exhibited hypertension-induced pathological changes. *ApoE/NOS3^−/−^* mice also inherited the dyslipidemia phenotype of *ApoE^−/−^* mice. The TC and LDL levels of adult *ApoE/NOS3^−/−^* mice were much higher than those of WT mice of the same age and higher than those of ApoE^−/−^ mice of the same age. *ApoE/NOS3^−/−^* mice had more severe hepatic steatosis and atherosclerosis than ApoE^−/−^ mice. These pathological symptoms were similar to those observed in patients with clinically hypertensive atherosclerosis, and the established model was successful [35,36]. Notably, offspring of *ApoE/NOS3^−/−^* mice were difficult to obtain after they were fed a high-fat diet, but they had a normal reproductive performance under normal diet conditions. We hypothesized that a high fat content in mice would damage embryonic development [37,38,39]. The reproductive status of this model is not particularly affected; therefore, *ApoE/NOS3^−/−^* mice have a potential application as a new model of hypertensive atherosclerosis. Furthermore, we observed that *ApoE/NOS3^−/−^* mice had severe renal lesions at 12 weeks of age, and clinically hypertensive atherosclerotic renal damage was not as severe. In *NOS3^−/−^* mice, the literature has reported that the diuretic response to a water load might be impeded, resulting in impaired renal salt excretion [40,41,42]. Therefore, we hypothesized that the renal lesions exhibited by *ApoE/NOS3^−/−^* mice were possibly due to a combination of hypertension and reduced salt sensitivity. We plan to conduct in-depth research on this aspect in the future. The focus of this study was to develop an atherosclerosis model with hypertension, and there have been few comparisons between *ApoE/NOS3^−/−^* and *NOS3^−/−^* mice. Compared with *ApoE^−/−^* mice, *ApoE/NOS3^−/−^* mice showed more severe atherosclerosis, suggesting that hypertension may aggravate the process of atherosclerosis, and the underlying mechanism needs to be studied further [43]. Considering that hypertensive atherosclerosis is a disease often observed in middle-aged and elderly individuals, this study did not compare the differences in related indicators between juvenile *ApoE/NOS3^−/−^* mice, WT mice, and *ApoE^−/−^* mice of the same age [44,45].

The symptoms observed in *ApoE/NOS3^−/−^* mice are caused by the loss of function of the *ApoE* and *NOS3* genes, and the etiology of hypertension and atherosclerosis is often complicated and unclear in clinical practice [46,47,48]. Therefore, the pathogenesis of this model may be inconsistent with that of humans and may not fully reflect human diseases. Hence, further research is required in this regard.

## 5. Conclusions

We established *ApoE* and *NOS3* double knockout mice with typical clinical features of hypertension and atherosclerosis. This model can be useful in the study of cardiovascular-related diseases.

## Figures and Tables

**Table 1:** The blood pressure of WT, *ApoE^−/−^*, *NOS3^−/−^*, and *ApoE/NOS3^−/−^* mice.

	WT	ApoE^−/−^	NOS3^−/−^	ApoE/NOS3^−/−^
Blood pressure (mmHg)	105.25 ± 7.74	113.00 ± 7.15	130.25 ± 6.71 **	133.00 ± 3.85 **

The mice were all 16 weeks old and were fed a basal diet. *NOS3^−/−^* and *ApoE/NOS3^−/−^* groups were compared with those of the WT group, respectively. Data are presented as the means ± SEM. *n* = 8, ** *p*-value < 0.01.

**Table 2:** The blood lipid levels of WT, *ApoE^−/−^*, and *ApoE/NOS3^−/−^* mice with a chow diet.

Items (mmol/L)	16 Weeks Old			24 Weeks Old		
	WT	ApoE^−/−^	ApoE/NOS3^−/−^	WT	ApoE^−/−^	ApoE/NOS3^−/−^
**TC**	2.49 ± 0.42	12.86 ± 1.92 **	13.45 ± 1.52 **	2.35 ± 0.31	15.78 ± 2.99 **	17.35 ± 1.07 **
**TG**	0.56 ± 0.12	0.66 ± 0.09	0.69 ± 0.16	0.77 ± 0.35	0.92 ± 0.28	0.86 ± 0.26
**HDL**	1.29 ± 0.29	1.21 ± 0.19	1.49 ± 0.31	1.31 ± 0.27	1.85 ± 0.31 **	1.69 ± 0.26 *
**LDL**	0.27 ± 0.07	1.88 ± 0.42 **	2.45 ± 0.59 **	0.36 ± 0.06	4.21 ± 1.35 **	5.90 ± 0.44 **

TC, total cholesterol; TG, triglyceride; HDL, high-density protein; LDL, low-density lipoprotein. ApoE^−/−^ and ApoE/NOS3^−/−^ groups were compared with those of the WT group, respectively. Data are presented as the means ± SEM. *n* = 8, * *p*-value < 0.05, ** *p*-value < 0.01.

**Table 3:** The blood lipid levels of WT, *ApoE^−/−^*, and *ApoE/NOS3^−/−^* mice with an obesogenic diet.

Items (mmol/L)	16 Weeks Old			24 Weeks Old		
	WT	ApoE^−/−^	ApoE/NOS3^−/−^	WT	ApoE^−/−^	ApoE/NOS3^−/−^
**TC**	3.44 ± 0.60	19.10 ± 2.14 **	27.54 ± 2.88 **	3.39 ± 0.27	21.32 ± 2.97 **	28.46 ± 4.73 **
**TG**	0.56 ± 0.08	0.65 ± 0.08	1.50 ± 0.82 **	0.65 ± 0.15	0.85 ± 0.20 *	1.31 ± 0.51 **
**HDL**	1.63 ± 0.41	2.08 ± 0.19 *	2.64 ± 0.47 **	1.83 ± 0.18	2.11 ± 0.21 *	3.33 ± 0.45 **
**LDL**	0.39 ± 0.09	5.65 ± 1.36 **	9.45 ± 1.93 **	0.41 ± 0.04	7.17 ± 1.39 **	13.84 ± 4.14 **

TC, total cholesterol; TG, triglyceride; HDL, high-density protein; LDL, low-density lipoprotein. ApoE^−/−^ and ApoE/NOS3^−/−^ groups were compared with those of the WT group, respectively. Data are presented as the means ± SEM. * *p*-value < 0.05; ** *p*-value < 0.01.

**Table 4:** Average litter size and weaned number of *ApoE^−/−^*, *NOS3^−/−^*, *ApoE/NOS3^−/−^* mice.

Strain	Average Litter Size (*n* = 8)				Average Weaned Number (*n* = 8)			
	Parity 1	Parity 2	Parity 3	Parity 4	Parity 1	Parity 2	Parity 3	Parity 4
**A**	5.25 ± 2.53	6.17 ± 2.26	5.75 ± 2.73	5.92 ± 2.27	5.08 ± 2.85	5.08 ± 2.44	5.50 ± 2.58	4.92 ± 2.45
**N**	5.20 ± 1.23	5.10 ± 1.91	5.50 ± 2.84	4.20 ± 1.20	4.80 ± 1.55	4.70 ± 1.49	5.10 ± 2.51	3.50 ± 1.08
**AN**	4.71 ± 2.39	4.88 ± 2.47	4.64 ± 2.21	4.53 ± 2.20	4.36 ± 2.75	3.71 ± 2.90	3.23 ± 2.71	3.53 ± 2.53

A, *ApoE^−/−^* mice; N, *NOS3^−/−^* mice; AN, *ApoE/NOS3^−/−^* mice. Data are presented as the means ± SEM.

## Data Availability

Not applicable.
